# Differential effects of Paclitaxel on dendritic cell function

**DOI:** 10.1186/1471-2172-11-14

**Published:** 2010-03-19

**Authors:** Justin John, Mohammed Ismail, Catherine Riley, Jonathan Askham, Richard Morgan, Alan Melcher, Hardev Pandha

**Affiliations:** 1 Systems Biology Laboratory (UK) Ltd. Milton Park, Abingdon, Oxon, OX14 4SA, UK; 2 Oncology, Postgraduate Medical School, Daphne Jackson Road, University of Surrey, Guildford, GU2 7WG, UK; 3 Academic Unit of Oncology and the Cancer Research UK Clinical Centre, St James's University Hospital, Leeds, LS12 6AG, UK

## Abstract

**Background:**

The potential utility of dendritic cells (DC) as cancer vaccines has been established in early trials in human cancers. The concomitant administration of cytotoxic agents and DC vaccines has been previously avoided due to potential immune suppression by chemotherapeutics. Recent studies show that common chemotherapy agents positively influence adaptive and innate anti-tumour immune responses.

**Results:**

We investigated the effects of paclitaxel on human DC biology *in vitro*. DCs appear to sustain a significant level of resistance to paclitaxel and maintain normal viability at concentrations of up to 100 μmol. In some cases this resistance against paclitaxel is significantly better than the level seen in tumour cell lines. Paclitaxel exposure led to a dose dependent increase in HLA class II expression equivalent to exposure to lipopolysaccharide (LPS), and a corresponding increase in proliferation of allogeneic T cells at the clinically relevant doses of paclitaxel. Increase in HLA-Class II expression induced by paclitaxel was not blocked by anti TLR-4 antibody. However, paclitaxel exposure reduced the endocytic capacity of DC but reduced the expression of key pro-inflammatory cytokines such as IL-12 and TNFα. Key morphological changes occurred when immature DC were cultured with 100 μmol paclitaxel. They became small rounded cells with stable microtubules, whereas there were little effects on LPS-matured DC.

**Conclusions:**

The effect of paclitaxel on human monocyte derived DC is complex, but in the clinical context of patients receiving preloaded and matured DC vaccines, its immunostimulatory potential and resistance to direct cytotoxicity by paclitaxel would indicate potential advantages to co-administration with vaccines.

## Background

Dendritic cells (DC) are specialized antigen presenting cells that can initiate a primary immune response on encountering foreign antigens [1]. There has been much focus aimed at harnessing their potency in several clinical applications including cancer, infectious and inflammatory diseases [2-4]. However, there has been limited success in the treatment of many cancers using dendritic cell based immunotherapy [5]. DC are capable of ingesting dead and dying (apoptotic) tumour cells which potentially expose the DC to an array of tumour-associated antigens for processing and presentation to T cells via HLA class I and II pathways [6-8]. Whilst several methods for loading DC *ex vivo *with tumour antigens are currently used, including DNA, RNA, peptides and apoptotic tumour cells, the optimal approach has yet to be determined. The reasons why individual loading strategies may fail to successfully induce anti-tumour immunity are still not fully understood. However, an understanding of the natural mechanisms by which DC acquire tumour antigens and mature *in situ *has resulted in the improvement of several DC-based immunotherapy strategies.

It has been proposed that *in situ *destruction of tumour cells using chemotherapy, radiotherapy or other physical methods releases suitable antigenic material which can lead to enhanced antigen acquisition and stimulation of an immune response [9]. The chemotherapeutic agent paclitaxel (Paclitaxel) induces cancer cell death by promoting the polymerisation of tubulin, thereby causing cell death and apoptosis by disrupting the normal microtubule dynamics required for cell division [10]. Paclitaxel has been shown to be highly immunosuppressive at cytotoxic doses. There is clinical evidence to show that systemic administration of cytotoxic compounds such as paclitaxel can have a detrimental effect on the number of systemic DC [11].

However, at lower concentrations there is also evidence to suggest that paclitaxel may be immunostimulatory which may contribute to the overall antitumour effects in the clinical setting [12-17]. Several murine cancer models have demonstrated that combined chemotherapy and DC-based immunotherapy can lead to complete tumour regression in contrast to partial regression in response to each element used individually [18].

The maturation status of DC is a key factor required for the induction of a specific immune response and is reliant on the presentation of antigens by fully mature DC. Paclitaxel has been shown to interact with TLR-4, a know receptor for lipopolysaccharide (LPS), on murine myeloid cells [19,20]. However, the effects of paclitaxel on DC maturation remain to be clarified.

The aims of this study were to evaluate the immunomodulatory effects of paclitaxel applied to *ex vivo *generated DC in terms of phenotype, function and cytokine expression. The findings of this study may have important implications in treating malignancies with chemotherapy and concomitant administration of *ex vivo *DC.

## Results

### MTS assay of melanoma cell and DC mitochondrial activity

**Figure 1:**
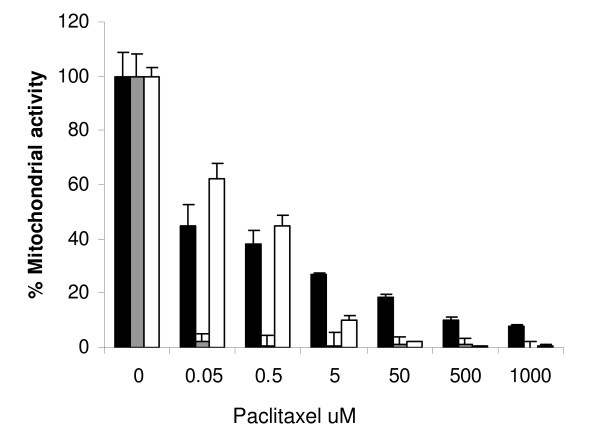
**Paclitaxel inhibits mitochondrial activity in tumour cell lines and DC as assessed by MTS/PMS assay**. Tumour cell lines MJT-3 (black squares), MCF7-pR (grey squares) and DC (white squares) were incubated with paclitaxel for 2 h before being washed and returned to culture for 48 h. Sensitivity was determined by the addition of MTS/PMS for triplicate samples ± SD. Each experiment was repeated 3 times and DC from 3 individual donors evaluated. Representative histograms from one experiment are shown. At lower doses (up to 0.5 μmol) of paclitaxel, the DC are more resistant than tumour cells (p value = 0.003). DC were more resistant overall than MCF7-pR.

In clinical practice chemotherapy exposes both tumour cells and the cells of the immune system to the cytotoxic potential of the drug. A common target in either cell type is cytoplasmic mitochondrial function. The potency for inhibition of this activity can be reliably demonstrated using the MTS/PMS assay. The human breast adenocarcinoma cell line MCF7-pR has been shown to be sensitive to paclitaxel both *in vitro *and *in vivo *in tumour models [21]. The cytoxicity of this and other chemotherapeutic compounds on the newly established melanoma cell line MJT-3 has been previously demonstrated in our laboratory (unpublished data). Figure 1 shows that both MCF7-pR and MJT-3 cell lines are sensitive to paclitaxel over a wide range on concentrations from 0.05 to 1000 μmol. DC were less sensitive to paclitaxel than MCF7-pR, with around 60% difference in mitochondrial activity, and marginally more sensitive than MJT-3 tumour cell lines with a difference of nearly 20% when treated with 0.05 μmol.

### Correlation of DC viability by TB and PI staining

**Figure 2:**
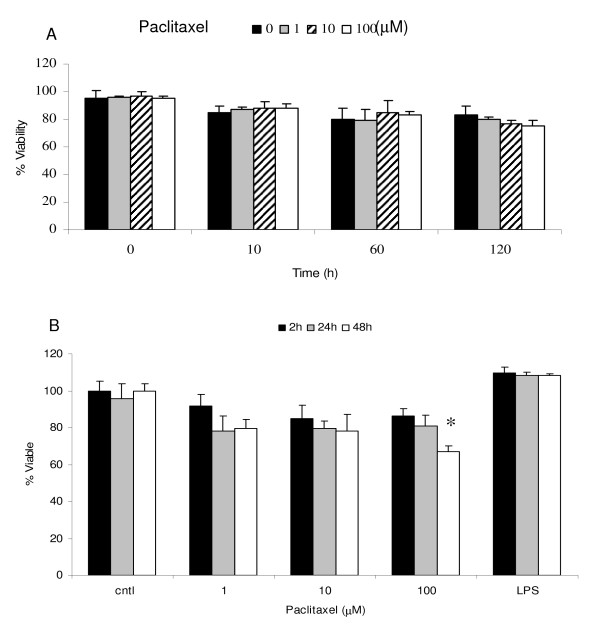
**(A) DC viability after exposure to paclitaxel**. Day 7 DC were incubated with paclitaxel at 1-100 μM for 2 h, washed and returned to culture for up to 120 h (5 days). Viability was assessed by trypan blue (and propidium iodide (PI) dye exclusion, data not shown). Representative data from one of 3 individual donors are shown. No deleterious effect on survival was seen, **(B)**. DC were exposed to paclitaxel at 1-100 μM for 2, 24 or 48 h and viability assessed at the end of 48 h by PI dye exclusion. Short-term exposure of DC to paclitaxel had no effect on viability, however, 100 μM paclitaxel for 48 h did induce a significant loss in viability (* P < 0.05).

DC were incubated with paclitaxel as in the MTS/PMS assay but were analysed for viability and membrane integrity by employing TB dye exclusion by microscopy and PI exclusion by flow cytometry (data not shown). As can be seen in figure 2A, DC viability by PI exclusion was maintained even when the DC were exposed to Paclitaxel for 2 h at concentrations up to 100 μM and returned to culture for up to 5 days.

At least 80% viability was maintained when DC were continuously exposed to paclitaxel for 24 h and 48 h at concentration below 100 μM. However, when DC were exposed to paclitaxel at 100 μM for 48 h there is a significant decrease of viability to around 60%, figure 2B. In comparison, DC exposed to LPS for the same duration of the experiment maintained their viability, consistent with reports of LPS inducing a survival signal to DC [22].

### Phenotypic changes of DC treated with Paclitaxel

The resistance of DC to paclitaxel at concentrations up to 100 μM was also reflected in surface marker expression by flow cytometry. As outlined in Table 1 the addition of paclitaxel led to the dose dependent increase in the expression of HLA class II molecules (Table 1) i.e. the MFI for the cells treated with 1, 10 or 100 μM was 690, 888 1109 respectively. The expression levels of Class II on paclitaxel-treated DC at 100 μM were equivalent to that induced by LPS. Higher concentrations of paclitaxel (1 mM) did not permit analysis of class II expression due to DC viability being too low for analysis (data not shown). There were no significant effects on co-stimulatory molecule expression or on CD209 or CD197.

**Table 1:** Expression of DC surface markers in response to paclitaxel.

			Taxol(μM)		
**Marker**	**No Drug**	**LPS**	**1**	**10**	**100**

HLA Class II	625	1265	690	888	1109

HLA Class I	93	274	95	85	87

CD40	4	7	6	6	7

CD80	40	95	57	61	51

CD83	5	22	4	5	4

CD86	18	71	27	26	33

DC were incubated with paclitaxel at various concentrations or LPS (1 μg/ml) for 2 h before being washed and returned to culture for a further 24 h. Surface expression of various molecules was assessed by flow cytometry as outlined in the *materials and methods*. The panel reports the delta MFI (the difference between isotype-matched mAb and specific mAb) mean fluorescence intensity (MFI). Data representative of 5 independent experiments.

### Enhanced DC stimulatory capacity at low dose paclitaxel

**Figure 3:**
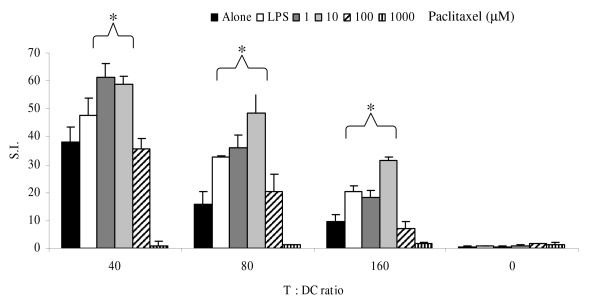
**Enhanced DC immunostimulatory capacity at low dose Paclitaxel**. Day 7 DC were treated with paclitaxel for 2 hours before being co-cultured with allogeneic T cells at different cell ratios in a 5 day MLR. The proliferation of the T cells was determined by the incorporation of [^3^H] thymidine and the data shown are the mean S.I. ± SD of triplicate samples, and is representative of 3 independent experiments using DC from 3 different donors. (* P < 0.05).

The immunostimulatory properties of Paclitaxel-treated DC were assessed by studying their ability to induce the proliferation of allogeneic T cells. The addition of paclitaxel at a concentration of 100 μM did not significantly alter the ability of DC to induce proliferation when compared to non-treated counterparts. Higher concentrations of paclitaxel (1 mM) totally abolished proliferation indicative of toxicity to the DC at the higher dose (figure 3). The stimulatory effect of 1 μM and 10 μM doses of paclitaxel on DC was significantly higher (p < 0.05) when compared to LPS matured DC and untreated controls. At a 80:1 T:DC ratio the biggest increase was seen with a 3-fold increase in proliferation with DCs treated with 10 μM and 2-fold increase with DCs treated with 1 μM. This enhancement in proliferation was maintained at all ratios of T cell:DC tested.

### Paclitaxel reduces receptor-mediated endocytosis

**Figure 4:**
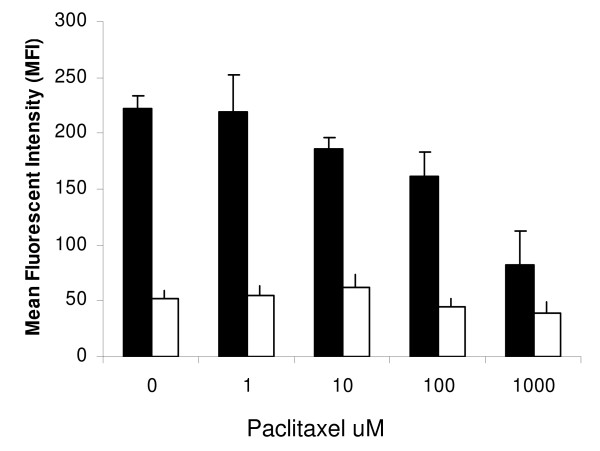
**DC exposed to paclitaxel have less endocytic function shown by uptake of FITC-conjugated dextran**. Control and paclitaxel treated DC were incubated with FITC-dextran for 2 h at either 37°C (black bars) or 4°C (white bars). DC were washed with ice cold PBS to remove unbound FITC-dextran prior to FACS analysis. Combined data from 3 separate donor experiments are shown. Increasing doses of paclitaxel significantly reduced active endocytosis compared to no taxol exposure (P < 0.05 with 10 μmol taxol exposure).

As already demonstrated, the exposure of DC to paclitaxel induced HLA class II expression but not other markers including the mannose receptor CD209 (data not shown). This was reflected by similar levels of dextran-FITC binding to the cell surface receptor of quiescent DC incubated at 4°C figure 4. However, during active endocytosis at 37°C an increase in the concentration of paclitaxel caused a decrease in the endocytic capacity of treated DC. At concentrations above 10 μM the level of uptake was significantly reduced by at least a 1.5-fold change in MFI when compared to untreated DC (P < 0.05 at 10 μmol), figure 4.

### Microarray analysis

The effect of paclitaxel and LPS on day 7 DC were examined by microarray. All microarray data was deposited in Array express (accession number E-MEXP-2465, experiment name Pandha DC taxol). Duplicate samples were compared. Fold changes in gene expression compared to untreated cells are shown in table 2. Only significant differences with p < 0.01 are shown. As expected the most significant effects were observed with LPS with induction of Th1 cytokine and chemokine gene expression, STAT1 activation and CD69, a marker of T cell activation. Other findings included an increase in cell cycle gene p21, FAS and FAS ligand, receptors such as ICAM1 and CXCR4, interferon alpha response genes IFI27 and NFκB. In contrast, paclitaxel reduced the expression of most of these genes apart from IL-1alpha. LPS induced TLR2 expression but resulted in marked reduction in TLR4 expression: paclitaxel was associated with a moderate reduction in expression of TLR2.

**Table 2:** Microarray analysis of DC exposed to LPS 1 μg/ml or paclitaxel 100 μM for 2 hours.

	Name	Accession number	Taxol	LPS
Cytokines	IL6	NM_000600	0.625	**13.75**
	IL1B	NM_000576	0.57	**21.4**
	IL10	NM_000572	0.14	**1.3**
	IL18	NM_001562	0.44	**4.77**
	IL1A	NM_000575	**2.33**	**2.5**
	TGFβ	NM_000660	0.5	0.43
	CXCl12	NM_199168	**1.428**	**42.3**
Cell cycle	CCND1	NM_053056	0.286	0.11
	p21	NM_000389	0.875	**3.125**
	ASK	NM_006716	0.833	0.417
	PCNA	NM_002592	0.6	**1.25**
Apoptosis	API4	NM_001012271	0.4	1
	FAS	NM_000043	0.667	**6.22**
	CASP8	NM_033356	**1.2**	0.45
	TNFRSF11B	NM_002546	0.1	**2.2**
	CASP3	NM_004346	0.7	**3.3**
	TNFα	NM_000594	0.4	**3**
	FASLG	NM_000639	0.375	**5.625**
Receptors	ICAM1	NM_000201	0.667	**3.77**
	TLR2	NM_003264	0.413	**3.45**
	TLR4	NM_003266	0.769	0.078
	CXCR4	NM_001008540	1	**2**

(Bold signifies upregulation)

Data shown is for samples where gene expression differed from untreated

cells (where P < 0.01). All microarray data was deposited in Array express; accession number E-MEXP-2465, experiment name Pandha DC taxol.

### Differential effect of paclitaxel on cytokine secretion by LPS-treated DC

**Figure 5:**
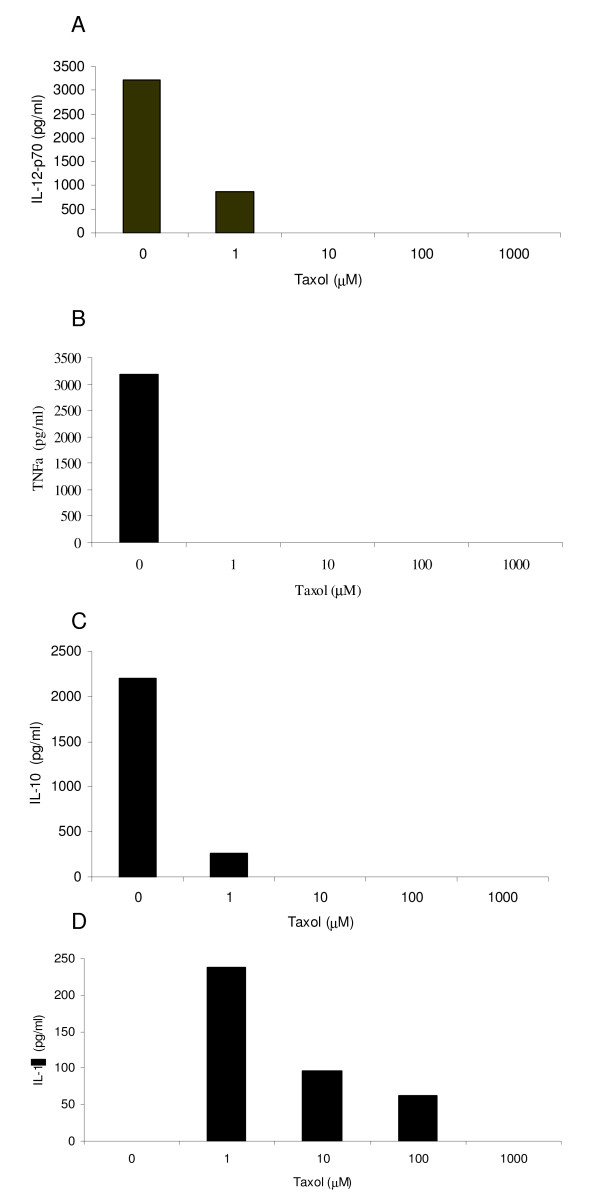
**Effects of Paclitaxel versus LPS on the ability of DC to produce inflammatory cytokines**. DC treated with LPS or paclitaxel for 2 hours were extensively washed and returned to culture for 24 h. Supernatants were collected and analysed by CBA for the presence of A; IL-12; B, TNFα; C, IL-10; D, IL-1β; EIL - 8. Overall, paclitaxel appeared to reduce cytokine secretion by DC compared to LPS; significant increases of IL-1β secretion were seen at lower doses of paclitaxel.

DC produced an array of inflammatory cytokines in response to LPS stimulation. In keeping with the microarray findings, the addition of paclitaxel inhibited the expression of IL-12p70, TNFα, IL-10, IL-8 and IL-1β at all concentrations tested (Figure 5A-E)

### Paclitaxel induces actin remodelling of DC cytoskeleton

**Figure 6:**
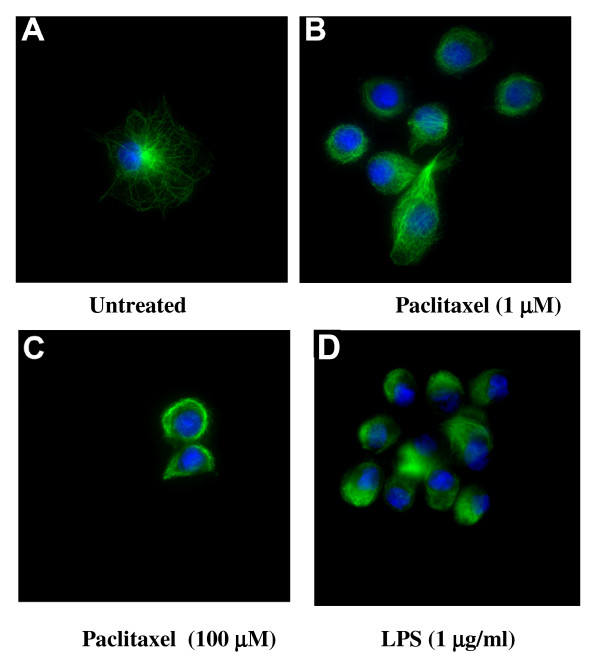
**Paclitaxel induced alterations to DC morphology and cytoskeletal organisation similar to LPS**. DC were adhered to fibronectin-coated coverslips prior to treatment with LPS +/- Paclitaxel for 2 h. The cells were extensively washed before being stained for microtubule arrangements and analysis by microscopy. Representative figures from 4 experimental repeats are shown. A, non-treated DC; B; 1 μM paclitaxel; C, 100 μM paclitaxel; D, 1 μg/ml LPS. In keeping with its known action, exposure to paclitaxel resulted in microtubule stabilisation but marked changes in DC morphology reduction in cell size, rounding and loss of dendrites.

In the absence of LPS or paclitaxel immature DC were characterised as being relatively large, irregular shaped bodies with a readily visible dense network of microtubules often focusing around the centrosome, figure 6(A). The treatment of DC with 1 μM paclitaxel resulted in distinct alterations to both DC morphology and microtubule distribution. Many of the DC became rounded with evidence of microtubule stabilisation, figure 6(B). The changes in DC cytoskeleton organisation were even more dramatic with 100 μM paclitaxel where all cells appeared small, rounded with microtubules that were no longer focused around the centrosome figure 6(C). Maturation in response to 1 μg/ml LPS also induced a rapid reduction in the size of the DC to smaller rounded cells with many forming small clumps figure 6(D). Again, the microtubules were less distinct and paralleled than observed for the paclitaxel treated DC. Treatment of LPS matured DC with various concentrations of paclitaxel had no distinguishable effects on morphology or microtubule stabilisation when compared to LPS alone or high dose paclitaxel treated DC (data not shown).

### Effects of paclitaxel are not mediated via TLR-4 binding

**Figure 7:**
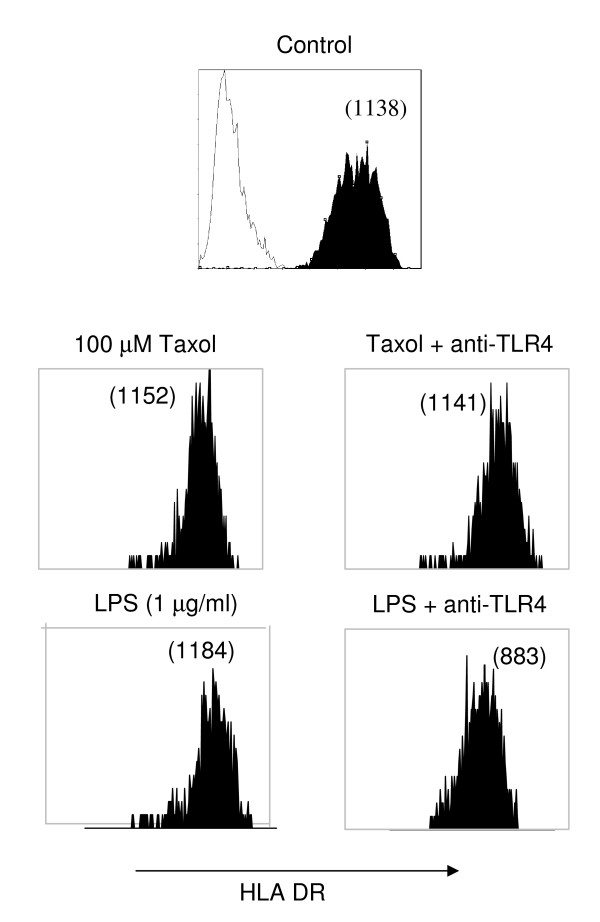
**Upregulation of DC class II expression by paclitaxel is not mediated via TLR-4**. DC were cultured for 2 h with LPS or paclitaxel in the presence or absence of anti-TLR-4 Abs prior to washing and reculture for a further 24 h. Anti-TLR4 antibody did reduce class II expression after exposure to LPS, but not to paclitaxel at high dose. Numbers in parenthesis represent MFI of class II detection. Data is representative of 3 independent experiments from 3 donors.

We have demonstrated that the treatment of DC with paclitaxel induces a dose dependent upregulation of surface HLA-class II expression. In order to determine whether this is mediated through the binding of TLR-4 we employed a blocking antibody against the TLR-4 molecule. Whilst LPS and paclitaxel induced the upregulation of surface class II molecules, blocking of TLR-4 with a specific monoclonal antibody only partially inhibited the response to LPS, figure 7.

## Discussion

Several chemotherapeutic agents have been shown to have dose dependent immunostimulatory activity [23]. However, there is conflicting data to suggest that these compounds are also capable of inhibiting the generation and maturation of cells capable of antitumour effects, especially in cancer patients who are already debilitated by their disease [24]. Paclitaxel is a widely used cytotoxic agent which targets microtubules and induces cellular death via apoptosis. The doses of paclitaxel under evaluation in this study reflect clinical practice: paclitaxel disappearance from plasma after intravenous infusion is biphasic; half-lives of the first and second phases occur approximately 0.3 and 8 hours, respectively. The peak plasma concentration with a high dose such as 275 mg/m^2^, occurs immediately post-infusion, and is approximately 8 μM [25]. We have demonstrated that, at similar plasma levels as in clinical practice, the mitochondrial function of human DC is less susceptible to inhibition by paclitaxel when compared to cancer cell lines. This is in accordance with data published on murine DC which were more resistant to paclitaxel compared to the murine melanoma cell line B16 [26]. The same report demonstrated that there was no induction of necrosis or apoptosis in murine DC treated with paclitaxel for 24 hours. A recent publication indicated significant effects of paclitaxel on DC at a dose range of 1-5 μM albeit on murine DCs [27]. We found, by two different methods, that paclitaxel at 100 μM does not induce either immediate cellular death or reduce DC viability for up to 5 days in culture. In contrast, it has been reported that human DC can undergo apoptosis following incubation with Paclitaxel at concentrations above 10 μM when continually exposed for 24 hours but these cells still maintaining mitochondrial function [28].

One possible mode of action by which chemotherapeutic agents may be immunomodulatory is by the induction of DC maturation by the upregulation of key molecules, especially those involved in antigen presentation such as HLA class II. We found that paclitaxel selectively enhanced the expression of HLA class II on DC in a dose dependent manner. Interestingly, the upregulation of class II did not correlate with allo-stimulatory activity. Enhancement of the allo-stimulatory activity was observed at low doses of paclitaxel which was superior to LPS activated DC. This would suggest that factors other than antigen presentation molecules are crucially affected by treatment with higher doses of paclitaxel. Low concentrations of paclitaxel have been shown to have a cytostatic effect on cells by effecting both cellular mitochondrial membrane and reducing potential. However, the apoptotic pathway is stopped upstream of mitochondria permeabilization and therefore does not lead to cell death[29]. The interactions of MHC class II and the mitochondria are central during the induction of mature DC death via cross linking of the surface molecules [30]. The expression of MHC class II in blood mononuclear cells is also associated with the presence of Myosin-V which is itself frequently associated with the central microtubules [31]. Therefore, alteration in MHC class II expression may be due to either paclitaxel induced disruption of mitochondrial function or the effect on microtubule arrangement via the co-localization with Myosin-V.

A reduction in the endocytic function of DC was also observed in response to paclitaxel despite no overall change in the expression of the specific surface receptor CD209. Phagocytosis of *Listeria monocytongenes *by macrophages has been shown to be dependent on functional actin filaments. Depolymerising drugs such as colchicine and nocodazole greatly reduced the cells ability to phagocytose *L. monocytogenes *while the stabilization of microtubles with paclitaxel had no significant effect on uptake [32]. Paclitaxel may therefore inhibit receptor mediated endocytosis by preventing mitochondrial activity from facilitating this active process. We have demonstrated that paclitaxel was very potent at altering both DC morphology and microtubule organization.

Paclitaxel treatment in patients results in the release of a spectrum of pro-and anti-inflammatory cytokines, but most significantly IL-10, IL-8 and IL-6 [33]. Microarray analysis of DC after paclitaxel exposure showed downregulation of most cytokine gene expression; the overall effects of paclitaxel appeared to be anti-inflammatory. Cytokine analysis from supernatants reflected these findings. In contrast, as expected, LPS effects on DC were profoundly pro-inflammatory. Of note also was the reduction in IL-12 production after exposure to paclitaxel suggests that paclitaxel-treated DC may induce a Th2 (or Th17) cell bias with implications regarding their use therapeutically. Similarly, the lack of chemokine secretion after paclitaxel exposure implies a reduction in DC migration to lymph nodes thereby reducing the possibility of priming naive T cells in a DC therapy context.

The reduction of mitochondrial potential in DC responding to glucocorticoid has been shown to inhibit DC maturation and IL-12 production [34]. As with MHC class II expression, depolarisation of the microtubule network can severely inhibit DC production of IL-12 in response to a TLR-4 agonist due to the disruption of intracellular TLR-4 [35]. Therefore, yet again paclitaxel-induced disruption of mitochondrial activity or microtubule arrangement may explain the inhibition of cytokine production.

Paclitaxel has been shown to mimic LPS activation of murine macrophages in a cell cycle-independent manner. Stimulation of these primary cells with paclitaxel is a TLR-4 and MyD88- dependent pathway which augments the release of TNF and nitric oxide [36]. TLR-4 has been shown to physically associate with the MD-2 and it is this molecule that confers LPS-responsiveness on TLR-4. The species-specific activity of paclitaxel is determined by murine MD-2 and not human MD-2, suggesting that MD-2 is the crucial element for recognition [37]. However, both we and others have demonstrated that human and murine myeloid cells respond to paclitaxel in a TLR-4 independent manner.

## Conclusion

In conclusion, this study has increased our understanding of the effects of a potentially immunomodulatory chemotherapeutic agent on the modulation of DC. Paclitaxel has been shown to both enhance and inhibit many functional capabilities of DC via inhibiting mitochondrial function and microtubule polymerization. Our preliminary data suggests that these mechanisms may occur in the absence of signaling through TLR-4. In the clinical scenario, overall, we would anticipate that concomitant administration of paclitaxel would enhance DC-based vaccine through upregulation chemokine expression and increased allostimulatory activity of DC although the precise time of administration of vaccine and paclitaxel need to be carefully considered in view of paclitaxel's rapid distribution post-infusion. Combined paclitaxel and DC-based vaccines are currently under investigation in a murine model.

## Methods

### Antibodies

The following antibodies were purchased from Serotec, Oxford, UK; HLA-ABC FITC HLA-DR,-DP,-DQ FITC; CD80 PE; CD86 PE, CD40 PE and anti-tubulin. The following antibodies were purchased from Becton Dickinson, Oxford, UK; CD83 FITC, CD197 PE and CD209 PE. The Alexa 488 conjugated anti-rat secondary antibody was purchased from Molecular Probes, Paisley, UK. Anti-TLR4 blocking antibody was purchased from Axxora, Nottingham, UK.

### Cell lines

The MCF7-pR human breast cancer cell line (a gift from Dr. Kay Colston, St George's University of London, UK) was grown in culture medium (CM) RPMI 1640; 10% FCS; 1% Penicillin/Streptomycin and 2 mM L-Glutamine all purchased from Sigma-Aldrich, Dorset, UK) and incubated at 37°C in a humidified incubator at 5% CO_2_. The melanoma cell line MJT-3 was previously established in our laboratory from a brain metastasis of a 45 yr old female patient [38] and cultured as outlined for MCF7-pR.

### Generation of monocyte-derived dendritic cells (DC)

Immature DC were prepared from peripheral blood mononuclear cells (PBMCs) isolated from buffy coats purchased from the blood bank. A minimum of 3 different buffy coats were used for each experiment. PBMCs were incubated with anti-CD14 microbeads (Miltenyi BiotecBergish Gladbach, Germany) as specified in the manufacturer's instructions. The isolated monocytes were resuspended at 10^6 ^cells/ml in medium supplemented with 10% human AB serum, 100 ng/ml GM-CSF (Leucomax; Novartis, Camberley, UK) and 50 ng/ml IL-4 (Peprotech EC Ltd, London, UK), cultured in tissue culture flasks. On day 7 the cells were defined as immature DC with low/intermediate HLA-DR expression

### *In vitro *cell drug-sensitivity assay

MCF7-pR and MJT-3 cells were plated in 96 well flat bottom plates at 5 × 10^4 ^cells/well in CM. Paclitaxel (Bristol Myers Squibb, Princeton, US) was serially diluted in CM and added to the cells to give a final concentration range of 0-100 μM. Day 7 DC were plated at 10^5 ^cells/ml in CM. The cells were incubated for 2 h and then washed twice by centrifugation with 200 μl fresh CM to remove the presence of the drug. The cells were allowed to recover in fresh medium for a further 48 h before the addition of MTS/PMS (Promega, Madison, USA). Optical density (O.D.) at 490 nm was measured after a further 2 h incubation using a Dynex technologies MRX-II plate.

The percentage cell viability was calculated as;

% Viability = 100 × (absorbance of cells incubated with drug containing medium)/(absorbance of cells incubated with medium alone).

### DC viability assessment

DC viability was assessed after 48 h post incubation in the presence of paclitaxel at various concentrations for 2, 24 or 48 h of the treatment. At the end of the 48 h the cells were harvested, washed twice and resuspended in PBS for TB enumeration by light microscopy or PBS containing PI (Sigma-Aldrich) for analysis by flow cytometry. Viable cells were verified and enumerated depending on their ability to exclude TB or PI. The percentage of viable DC was calculated as outlined above.

### Direct surface staining for immunophenotyping

DC were harvested and washed in wash buffer (PBS containing 0.5% BSA and 0.1% sodium azide, all purchased from Sigma-Aldrich). Cells were labeled with the relevant fluorochrome-conjugated antibody at the concentration recommended by the manufacturer for 30 min on ice in the dark. DC were also incubated with an irrelevant isotype-matched control antibody to compensate for non-specific binding. The cells were then washed in wash buffer and the cell pellet fixed with 200 μl CellFix (Becton Dickinson). Samples were either analysed immediately or within 24 h (stored at 4°C in the dark) on a flow cytometer. Routinely 10,000 events were collected with dead cells and debris being gated out on the basis of their light scatter properties.

### Mixed Lymphocyte Reaction (MLR)

The stimulatory function of the drug-treated DC was assessed by their ability to induce proliferation in allogeneic non-adherent PBMCs. Day 7 DC were incubated in the presence of paclitaxel for 2 h as previously described. The cells were washed, resuspended in CM and graded numbers of DC co-cultured with 10^6 ^allogeneic non-adherent PBMCs. Proliferation was measured on day 5 following 18 h of pulsing with 1 μCi [^3^H]-Thymidine per well. Mean values of triplicates were measured and expressed as stimulation indices (SI).

SI = (mean counts per minute for test sample)/(mean counts per minute of background)

### Receptor-mediated endocytosis

The endocytic capacity of DC was determined as followed. Briefly, DC were treated as before with various concentrations of paclitaxel for 2 h prior to incubation at 4°C or 37°C for 10 min in CM to equilibrate the temperature. Dextran-FITC (40,000 MW- Sigma-Aldrich) was added to each sample and incubated for a further 60 min at the same temperature. Washing the cells twice in ice cold PBS quenched the endocytic activity and removed any free dextran-FITC. Cells were fixed in 200 μl of CellFix and routinely 5000 cells were analysed by flow cytometry. Surface only binding of dextran-FITC was deemed to occur when the DC were incubated at 4°C while endocytosis was judged to occur at 37°C.

### Measurement on cytokine production

IL-12p70, TNFα, IL-10 and IL-1β were analysed using the cytometric bead array (Becton Dickinson). Supernatants were collected from DC after treatment with LPS (1 μg/ml) or Paclitaxel for 2 h followed by re-culture for 24 h. The supernatants were frozen at -20°C until analysis.

### Actin-remodelling in DC

Coverslips were washed in 70% ethanol for 10 min, followed by brief washes in 95% and then 100% ethanol before air drying. Coverslips were inverted in 50 μg/ml fibronectin solution (Sigma-Aldrich) for 2 hours. Coverslips were rinsed extensively in PBS, placed in the wells of a tissue culture plate and covered with CM. Immature DC or LPS-matured DC were seeded on the fibronectin-coated coverslips for 1 hour. Cells were then treated with paclitaxel at a final concentration of 0-100 μM as required for 2 hours prior to fixation and immunocytochemistry. DC were washed twice in PBS before incubating in -20°C methanol for 5 min. Cells were blocked for 30 min in blocking buffer (PBS containing 1% non-fat dried milk) before incubation with anti-tubulin antibody for 1 hour. DC were washed in PBS before incubation with an Alexa 488 conjugated anti-rat secondary antibody for 1 h. Coverslips were washed before mounting on glass slides using Mowiol (Calbiochem, Nottingham, UK). Images were captured using a Zeiss Axiovert 200TV microscope connected to a Hammamatsu camera and imported into Imaris. Images were exported to and figures assembled in Adobe Photoshop.

### Blocking of TLR-4 receptor

DC were cultured with LPS (1 μg/ml) or paclitaxel (100 μM) for 2 h in the absence or presence of 4 μg/ml of a blocking antibody directed towards TLR-4. The cells were washed before being returned to culture for a further 24 h. Cells were assessed for the expression of HLA DR as outlined above.

### Microarray analysis of the effects of Paclitaxel and LPS on DC

Total RNA was extracted from day 7 DC which had been treated for 2 h with 1 μg/ml LPS or 100 μM paclitaxel for 2 h then washed and returned to culture for 24 h. This was used as a template to generate Cy3-labelled cRNA, using the Low RNA Input Linear Amplification Kit (Agilent). This was used as a probe on the Whole Human Genome Microarray (4 × 44 K) slide (Agilent). Slides were scanned using the Agilent scanner and data extracted using Feature Extraction Software 9.5.3 (Agilent). Subsequent data analysis was performed using GeneSpring GX software.

### Statistical analysis

The statistical significance of experimental data was evaluated using the Student's *t*-test where *P *< 0.05 was considered as statistically significant.

## Authors' contributions

JJ participated in experimental design and implementation. MI was involved in generation of DCs. CR carried out in vitro drug sensitivity assays. JA was involved in experimental techniques. RA was involved in statistical analysis. AM carried out a critical review of the manuscript. HP conceived the study and participated in its design and coordination and helped to draft the manuscript. All authors read and approved the final manuscript.
